# Lactational exposure to perfluorooctane sulfonate remains a potential risk in brain function of middle-aged male mice

**DOI:** 10.1186/s12576-024-00907-6

**Published:** 2024-03-05

**Authors:** Ayane Ninomiya, Izuki Amano, Hiraku Suzuki, Yuki Fujiwara, Asahi Haijima, Noriyuki Koibuchi

**Affiliations:** 1 Department of Integrative Physiology, Gunma University Graduate School of Medicine, 3-39-22, Showa-machi, Maebashi, Gunma 371-8511 Japan; 2 Department of Environmental Brain Science, Faculty of Human Sciences, Waseda University, 2-579-15, Mikajima, Tokorozawa, Saitama 359-1192 Japan

**Keywords:** Perfluorooctane sulfonate (PFOS), Alzheimer’s disease, Cognitive function, Aging

## Abstract

Perfluorooctane sulfonate (PFOS) exerts adverse effects on neuronal development in young population. Limited evidences have shown that early-life PFOS exposure holds a potential risk for developing age-related neurodegenerative diseases such as Alzheimer’s disease later in life. The present study investigated the effects of lactational PFOS exposure on cognitive function using one-year-old mice. Dams were exposed to PFOS (1 mg/kg body weight) through lactation by gavage. Male offspring were used for the behavior test battery to assess cognitive function. Western blot analysis was conducted to measure the levels of proteins related to the pathogenesis of Alzheimer’s disease. PFOS-exposed mice displayed a mild deficiency in social recognition. In the hippocampus, the expression of tau protein was significantly increased. These results underline a mild effect of developing PFOS exposure on cognitive function and neurodegeneration. The present study presents the long-lasting effects of PFOS in middle-aged period and warrants a potential aftermath.

## Background

Perfluorooctane sulfonate (PFOS) is classified to per- and polyfluoroalkyl substances (PFAS). Substances in PFAS family have an amphiphilic property and thus were once utilized as a surfactant for various industrial and commercial products including a firefighting foam, water- and oil-repellent carpets, or coated cookware [1]. Another chemical property of strong carbon–fluorine bonds makes PFAS hard to decompose and remain in the environment almost forever. As PFAS has accumulated in water, soil, air, and biota [2], humans could be easily exposed to such environmental chemicals. Indeed, PFAS has been detected in human serum and has exerted a series of toxic effects on the human body including the reproductive system, endocrine system, immune system, and so on [1].

In particular, the neurodevelopmental toxicity has been cautioned by previous studies both in humans and animals. Prenatal exposure to PFOS, is associated with lower cognitive performance in two-year-old children and poor nonverbal working memory in three and a half-year-old children [3, 4]. The cohort studies reported the negative association between maternal PFOS concentration and intelligence in preschool-aged and 7-year-old boys [5, 6]. In animal studies, we previously reported that the lactational PFOS exposure impairs learning and memory in male offspring [7]. Similarly, PFOS exposure during gestation and lactation reduces the spatial learning and memory abilities of the offspring, alongside the decrease in expression of proteins related to synaptic plasticity in the hippocampus [8]. The impairment in cognitive function can be explained by the PFOS effects on synaptic transmission and plasticity both in pre- and post-synaptic hippocampal neurons, as the gestational PFOS exposure inhibited long-term potentiation and suppressed paired-pulse facilitation of offspring [9]. We previously reported exactly the same finding in the cerebellum [10]. These findings clearly indicate that an early-life PFOS exposure gives a rise to the onset of neurodevelopmental retardation in child- or young adulthood.

Not only young population, but aging population is also highly vulnerable to PFOS toxicity, especially in terms of developing age-related neurodegenerative diseases. People living in the PFOS-contaminated area have higher odds ratio for developing Alzheimer’s disease and dying from Alzheimer’s disease-related causes compared to people living in the noncontaminated area [11]. Another screening study found the specific genomic profile pattern with a predisposition to Alzheimer’s disease in people who drink PFOS-contaminated water [12]. The animal studies with developmental PFOS exposure have observed the major pathological hallmarks of Alzheimer’s disease such as increased tau phosphorylation and protein expression as well as amyloid precursor protein and amyloid-$\beta$ 1–42 in adulthood [13, 14]. These findings could be a strong backbone to raise a hypothesis that PFOS may increase the expression of pivotal proteins in Alzheimer’s disease. However, these studies have a certain limitation of the experimental design such that they examined such biomarker proteins for Alzheimer’s disease at postnatal day (PD) 20 and 90, which are not sufficiently old to assess age-related neurodegeneration [13, 14]. Thus, further investigation is needed at the older ages.

To investigate a potential risk of PFOS exposure in developing aging-induced neurodegenerative diseases such as Alzheimer’s disease, the present study examined the effects of lactational PFOS exposure on cognitive function using one-year-old (PD > 365) aged male mice.

## Methods

### Chemical, animals, and treatment

PFOS (purity $\ge$ 98.0%) was purchased from Sigma Aldrich (St. Louis, MO, U.S.A.) and dissolved with 0.5% Tween 20 (Sigma Aldrich) in deionized water.

Under the council of the Animal Care and Experimentation Committee of Gunma University, the protocol for the animal experiment in the present study was approved (protocol number: 19-075). Female C57BL/6J mice were purchased from Japan SLC (Hamamatsu, Japan). On arrival, they were all pregnant at the gestational day 14 and immediately assigned to a single cage per each dam. PD 0 was considered as the day of delivery. The light cycle of the breeding room is 7:00–19:00. The temperature and humidity were kept 22–24 °C and 30–60%, respectively. Mice were allowed to reach food and water ad libitum.

After the delivery, PFOS was exposed to dams by gavage at the concentration of 1 mg/kg body weight during the lactation (PD 0- 14) as previously described [10]. Subsequently, the pups were exposed to PFOS through the breast milk. The equivalent volume of Tween 20 was given to the control dams for the same duration of period. Pups were isolated from their biological dams at PD 21. Only male offspring were used for the experiments at 1 year old (PD 365). The body weight was measured at six time points from PD 300 to PD 365. The whole brain weight was measured when sampling at PD 365. The use of female mice was avoided to exclude the hormonal effects of unstable estrous cycle and menopause in aging period ([15]). Nine mice were used for all behavioral tests in each group.

### Circadian locomotor activity test

The spontaneous locomotion with the circadian rhythm was tested using the method previously described [16]. A single mouse was put in the standard cage (23.5 cm $\times$ 16.5 cm $\times$ 13 cm) with food and water ad libitum. Mice were habituated in the novel cage for one day. For the next two days, the spontaneous locomotion was recorded in real time by an infrared sensor placed over the cage. Data were accumulated in an online data-acquisition system (NS-ASS01, O’hara & Co., Ltd., Tokyo, Japan).

### Open field test

Locomotor activity in an open field (45 cm $\times$ 45 cm $\times$ 20 cm) was analyzed as described before [17]. The activity was detected by 16 $\times$ 16 crossed infrared beams at intervals of 2.5 cm on the sides (LE 8811; Panlab, S.L.U., Barcelona, Spain) and tracked for 30 min by Acti-Track program (Panlab, S.L.U.). As a parameter, traveling distance and time in the center area were calculated.

### Visual discrimination test

The pairwise visual discrimination test was conducted to examine learning ability as previously described [7]. Prior to the experiment, the training session was held for five days to habituate mice to the test system. In the 30-min session, mice leaned to get a food pellet when they touched either of the paired panels displaying the same images in the chamber apparatus (O’hara & Co., Ltd). To motivate mice to get food, they were fasted over night before the training session. In the test session, two panels randomly switch to display the different images, horizontal or vertical stripes, during 50 trials. Mice were rewarded a food pellet only when they touched the correct panel (horizontal), but not rewarded when they touched the wrong panel (vertical). The percentage of choosing the correct panel out of 50 trials was calculated as % Correct. The test session was conducted for nine days.

### Object location test and object recognition memory test

Both of object location test and object recognition test were carried out to assess spatial memory in the same way as the young-adult mice did [7]. Following the 30-min habituation in an open arena (length, 30 cm; width, 30 cm; height, 39 cm), two identical objects were installed at the two diagonal corners at the end of one side of the arena (7.5 cm from each adjacent wall) and mice were given five minutes to explore them. Twenty-four hours later, mice were brought back to the arena where one of the objects was moved to a novel location (i.e., any one of the corners adjacent to the familiar one) and again given five minutes to explore (object location test). Another 24 h later, mice were introduced to a novel object which replaces one of the objects they saw one day ago and again given five minutes to explore (object recognition memory test). Test analysis was conducted using discrimination index as calculated by (time_novel_–time_familiar_)/(time_novel_ + time_familiar_) following the previous study [7].

### Elevated plus maze test

To investigate the anxiety-like behavior, the elevated plus maze test was conducted following the protocol in the previous study [10]. In brief, mice were placed in the center of the elevated pulse maze apparatus (LD-EPMM52515, LabDesign, Ibaraki, Japan). No habituation phase was preceded. The behavior was recorded by the video camera placed over the apparatus for 10 min. ANY-maze ver. 6.3 (Stoelting Co., Wood Dale, IL, U.S.A.) was used to measure the time spent in an open arm and closed arm.

### Light–dark chamber test

The anxiety-like behavior was examined using the light chamber (15 cm $\times$ 15 cm $\times$ 15 cm) with an access to the same-sized dark chamber through a 7 cm $\times$ 7 cm window as previously described [10]. ANY-maze ver. 6.3 (Stoelting Co.) was used to measure the time spent in the light chamber.

### Marble burying test

Following the previous study [10], a single mouse was placed in the cage (25 cm $\times$ 15 cm $\times$ 15 cm) with 20 marbles on a woodchip bedding (3 cm in depth). After 30 min, the number of unburied marbles was counted.

### Three-chamber social interaction test

The three-chamber social interaction test was conducted to examine the social behavior using the same protocol mentioned in the previous paper [10]. Mice were habituated to the three-chamber box (39 cm $\times$ 20 cm $\times$ 30 cm) for five minutes. In the session 1, a tested mouse was introduced to a novel mouse (“stranger 1”, age- and sex-matched) in a small box in the chamber 1. The box in the chamber 2 was empty. Time the tested mouse approached to each box was counted for 10 min. In the session 2, another novel mouse (“stranger 2”, age- and sex-matched) was placed in the chamber 1. Again, the approaching time to each chamber was counted for 10 min. The sessions were recorded by the video camera located over the apparatus and analyzed by ANY-maze ver. 6.3 (Stoelting Co.).

### Western blot analysis

The hippocampal tissues were collected from randomly selected five mice in each group at one year old. The whole brain was coronally sectioned using the rodent brain matrix (ASI Instruments, Inc., Warren, MI, U.S.A.) with a range of bregma—3.28 to 1.95 mm according to the hippocampal region of Allen Mouse Brain Atlas [18]. Total protein was extracted following the homogenization of the tissue samples using a Cell Lysis Buffer (Cell Signaling Technology, Danvers, MA, U.S.A.) containing protease inhibitors (cOmplete™, Mini Protease Inhibitor Cocktail, Roche, Basel, Switzerland). The protein concentration was measured by the Bradford protein assay (Bio-Rad Laboratories, Inc., Hercules, CA, U.S.A.) based on the instruction provided by the manufacture. Following the boiling with 2 × Laemmli Sample Buffer (Bio-Rad Laboratories, Inc.) for five minutes, 4 μg/10 μl protein samples were loaded to 4–20% Mini-PROTEAN TGX gel (Bio-Rad Laboratories, Inc.) for the electrophoresis. The separated products were transblotted to the Amersham™ Protran™ 0.2 μm nitrocellulose blotting membranes (GE Healthcare, Chicago, IL, U.S.A.). The membranes were blocked for one hour with Blocking One or Blocking One-P for the phosphorylated protein (NACALAI TESQUE, INC., Kyoto, Japan). The overnight incubation at 4 °C was carried out with the appropriate diluted primary antibodies for GSK3 $\beta$ (1:1000, #12456, Cell Signaling Technology), tau (1:1000, #4019, Cell Signaling Technology), phosphorylated tau (Ser404) (1:1000, #20194, Cell Signaling Technology), and GAPDH (1:1000, #2118, Cell Signaling Technology). After the rinse with Tris-buffered saline containing 0.1% Tween 20, horseradish peroxidase-conjugated anti-rabbit (1:10000, #7074, Cell Signaling Technology) or anti-mouse (1:10000, #7076, Cell Signaling Technology) were applied to the membranes for 1-h incubation at room temperature. After the rinse, Chemi-Lumi One Ultra (NACALAI TESQUE, INC.) was applied to the membrane for five minutes. Blotting images were obtained using LAS 4010 (GE Healthcare). GAPDH was used as a loading control. The untrimmed images of blotting membrane are provided as a supplemental file.

### Statistical analysis

Data analysis of all experiments was carried out using GraphPad Prism 10 for macOS (www.graphpad.com, San Diego, CA, U.S.A.). Two-way ANOVA was carried out for the body weight, locomotion activity test, open field test and the visual discrimination test. Unpaired *t*-test was conducted for the rest of experiments except brain weight, marble burying test and the protein analysis which were done by Mann–Whitney test. When *p* value was < 0.05, the result was considered as statistically significant. All data are expressed as the mean $\pm$ standard error of the mean (SEM).

## Results

### Body and brain weights

**Fig. 1:**
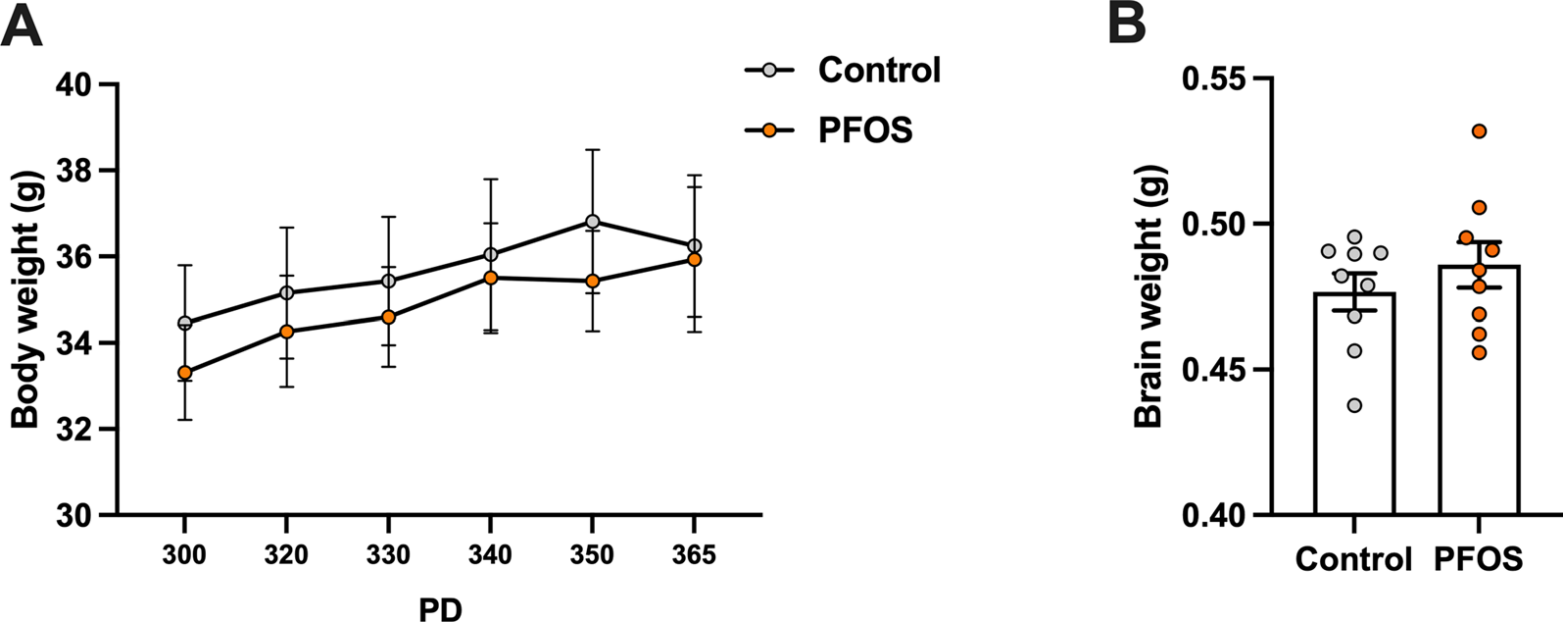
Body and brain weights. There were no differences in body weight (**A**) and brain weight at PD 365 (**B)** between control and PFOS group at aged period. Data are expressed as the mean $\pm$ SEM. *n* = 9 mice in each group

Body and brain weights were monitored in middle age to examine the long-lasting effects of developing PFOS exposure on body mass. There was no change in body weight from PD 300 to 365 between the control and the PFOS groups (*F* (1, 16) = 0.1805, *p* = 0.68, Fig. 1A). In addition, the whole brain weight of the PFOS group was comparable to that of the control group at PD 365 ([control] 0.48 $\pm$ 0.01 g, [PFOS] 0.49 $\pm$ 0.01 g, Mann–Whitney *U* = 33, *p* = 0.55, Fig. 1B). Overall, the lactational PFOS exposure has no effects on the body mass in middle-aged period.

### Locomotor activity in a home cage and an open field

**Fig. 2:**
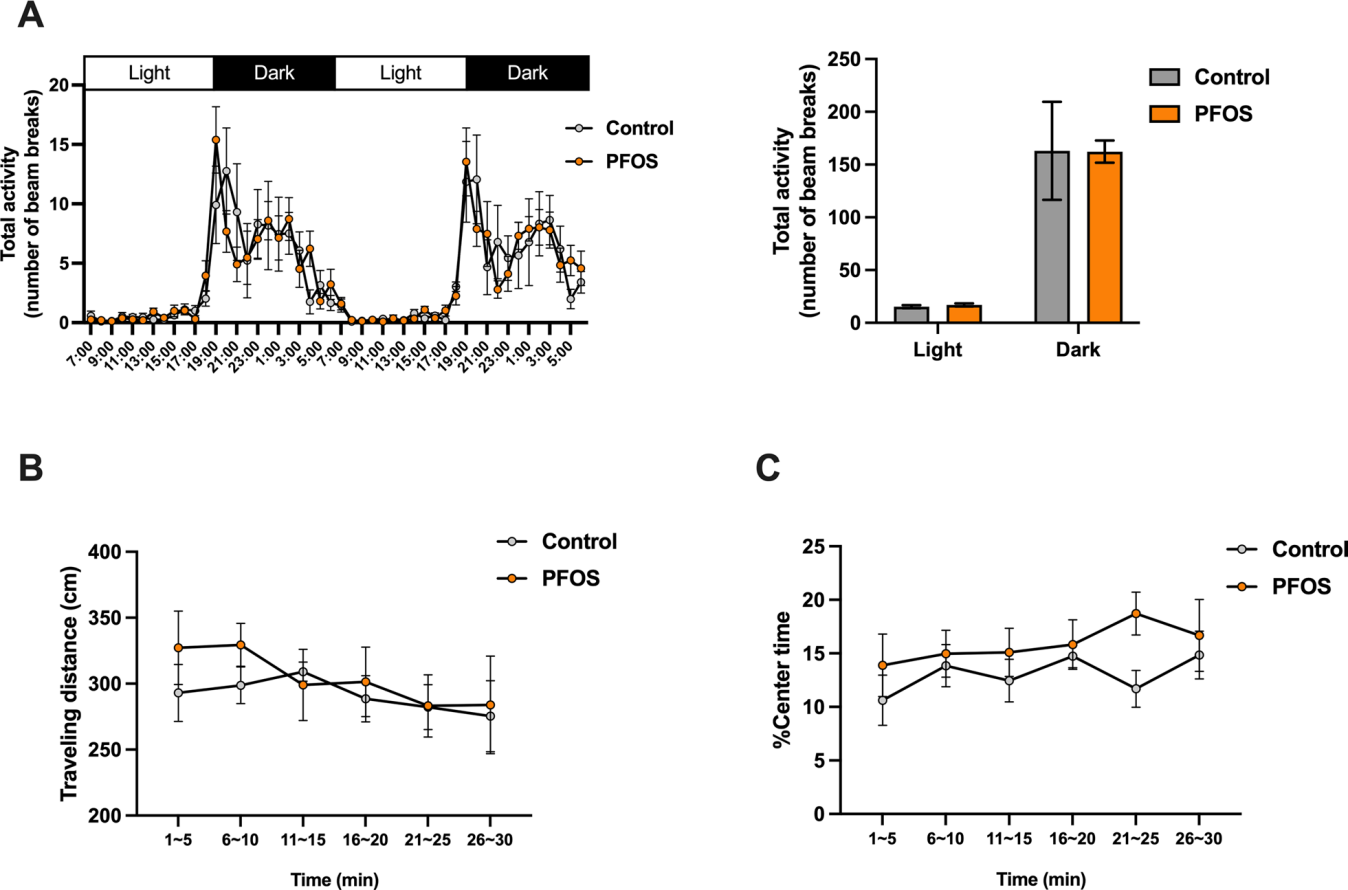
The locomotor activity in a home cage and an open filed. There was no difference in the home-cage activity level between control and PFOS mice during two-day monitoring (**A**). In an open filed, there were no differences in total travelling distance (**B**) and percentage of time spent in a center area (**C**). Data are expressed as the mean $\pm$ SEM. *n* = 9 mice in each group

To examine the basal activity level in a home cage and a novel environment, locomotor activity test and open field test were conducted, respectively. There was no difference in the locomotor activity in a home cage between the control and the PFOS groups both in the light and dark period (two days) (*F* (1, 16) = 0.00034, *p* = 0.99, Fig. 2A). In an open field, the traveling distance and time spent in a center area were not changed in the PFOS group (traveling distance: *F* (1, 16) = 0.304, *p* = 0.59, center time: *F* (1, 16) = 1.703, *p* = 0.21, Fig. 2B-C). These results indicate that the lactational PFOS exposure has no effects on the locomotor activity level in middle-aged period.

### Learning ability

**Fig. 3:**
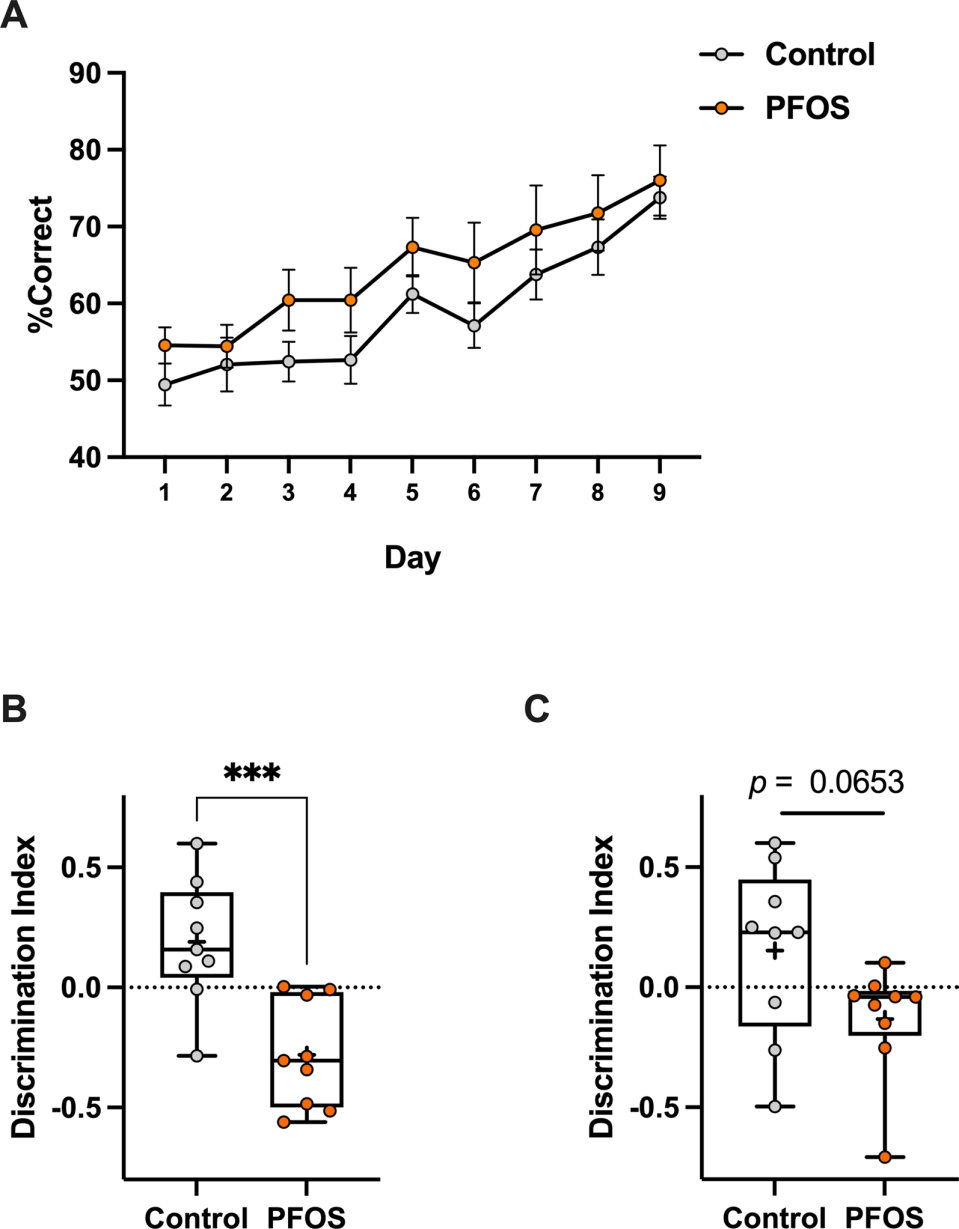
Learning ability in the pairwise visual discrimination test and spatial memory in object location test and object recognition memory test. There was no difference in the percentage of correct answer between groups for nine days performance (**A**). Long-term spatial memory was disrupted in PFOS-exposed mice in object location test (**B**). Object recognition memory was not impaired in PFOS-exposed mice (**C**). Data are expressed as the mean $\pm$ SEM. *n* = 9 mice in each group

Pairwise visual discrimination test was conducted to monitor learning ability. During the 9-day test period, the PFOS mice displayed an increase in the percentage of the correct answer (% Correct) day by day in the same manner as the control mice (*F* (1, 16) = 2.327, *p* = 0.15, Fig. 3A). There was no between-group difference in % Correct on each day. This result suggests that the lactational PFOS exposure has no effects on learning ability in middle-aged period.

### Spatial memory

Object location test and object recognition memory test were carried out to assess spatial memory. In object location test, PFOS mice showed significantly lower discrimination index compared to the control mice ([control] 0.1894 $\pm$ 0.08686, [PFOS] -0.2824 $\pm$ 0.07413, Mann–Whitney *U* = 4, *p* = 0.0005, Fig. 3B), indicating that they barely discriminated the novel location of the object. In object recognition memory test, PFOS mice tended to have lower discrimination index but not at the statistically significant level ([control] 0.1533 $\pm$ 0.1210, [PFOS] -0.1326 $\pm$ 0.07899, Mann–Whitney *U* = 4, *p* = 0.0653, Fig. 3C). Taken together, these results suggest that spatial memory is impaired in PFOS-exposed mice in the middle age.

### Anxiety-like behavior

**Fig. 4:**
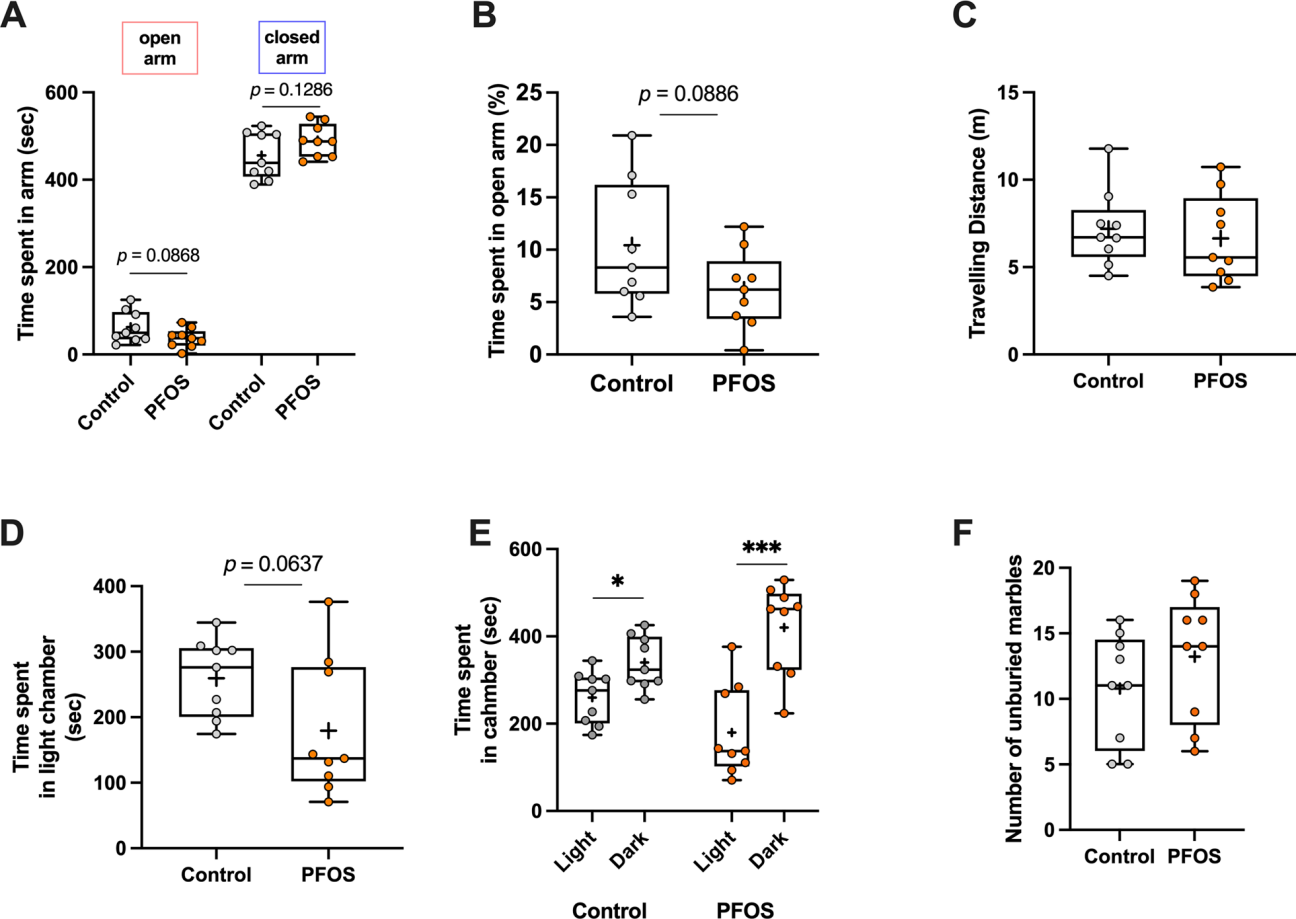
The effects of lactational PFOS exposure on anxiety-like behavior in middle-aged period. There was no significant difference in time spent in an open arm and a closed arm between groups in the session of the elevated pulse maze test (**A**). Also, time spent in an open arm did not differ between groups in percentage (**B**). The total traveling distance in an elevated pulse maze was comparable between groups (**C**). In the light–dark chamber test, no significant difference was detected in time spent in the light chamber between control and PFOS groups (**D**). Both groups increased time spent in a dark chamber at the statistically significant level (**E**). No difference in number of unburied marbles was observed in the marble burying test (**F**). * and *** indicate *p* < 0.05 and 0.001, respectively, by unpaired *t*-test. Data are expressed as the mean $\pm$ SEM. *n* = 9 mice in each group

Elevated pulse maze test, light–dark chamber test, and marble burying test were carried out to assess an anxiety-like behavior. For 10 min in the session in the elevated pulse maze test, there was no change in time spent both in an open arm and a closed arm between the control and the PFOS groups, although the PFOS group slightly tended to decrease the time spent in an open arm (open arm: [control] 62.63 $\pm$ 11.9 s, [PFOS] 37.13 $\pm$ 7.33 s, *t* = 1.824, *p* = 0.0868, closed arm: [control] 455.6 $\pm$ 17.75 s, [PFOS] 490.3 $\pm$ 12.47 s, *t* = 1.603, *p* = 0.13, Fig. 4A). Similarly, there was a tendency in the PFOS group to decrease the time spent in an open arm in percentage but without a statistical significance ([control] 10.42 $\pm$ 1.99%, [PFOS] 6.189 $\pm$ 1.22%, *t* = 1.813, *p* = 0.0886, Fig. 4B). The total traveling distance was not changed between groups ([control] 7.196 $\pm$ 0.73 m, [PFOS] 6.644 $\pm$ 0.83 m, *t* = 0.5016, *p* = 0.62, Fig. 4C). In the light–dark chamber test, PFOS tended to decrease the time spent in light chamber but with no significant difference ([control] 259.6 $\pm$ 20.03 s, [PFOS] 179.6 $\pm$ 34.75 s, *t* = 1.993, *p* = 0.0637, Fig. 4D). The time spent in the dark chamber was significantly increased in the both groups (control: [light] 259.6 $\pm$ 20.03 s, [dark] 340.4 $\pm$ 20.03 s, *t* = 2.855, *p* = 0.012, PFOS: [light] 179.6 $\pm$ 34.75 s, [dark] 420.4 $\pm$ 34.75 s, *t* = 4.898, *p* = 0.001, Fig. 4E). Finally, the marble burying test revealed no differences in the number of unburied marbles between groups ([control] 10.78 $\pm$ 1.402, [PFOS] 13.22 $\pm$ 1.588, Mann–Whitney *U* = 25.5, *p* = 0.1943, Fig. 4F). Taken together, there was no obvious effects of PFOS on the anxiety-like behavior in middle-aged period.

### Social recognition

**Fig. 5:**
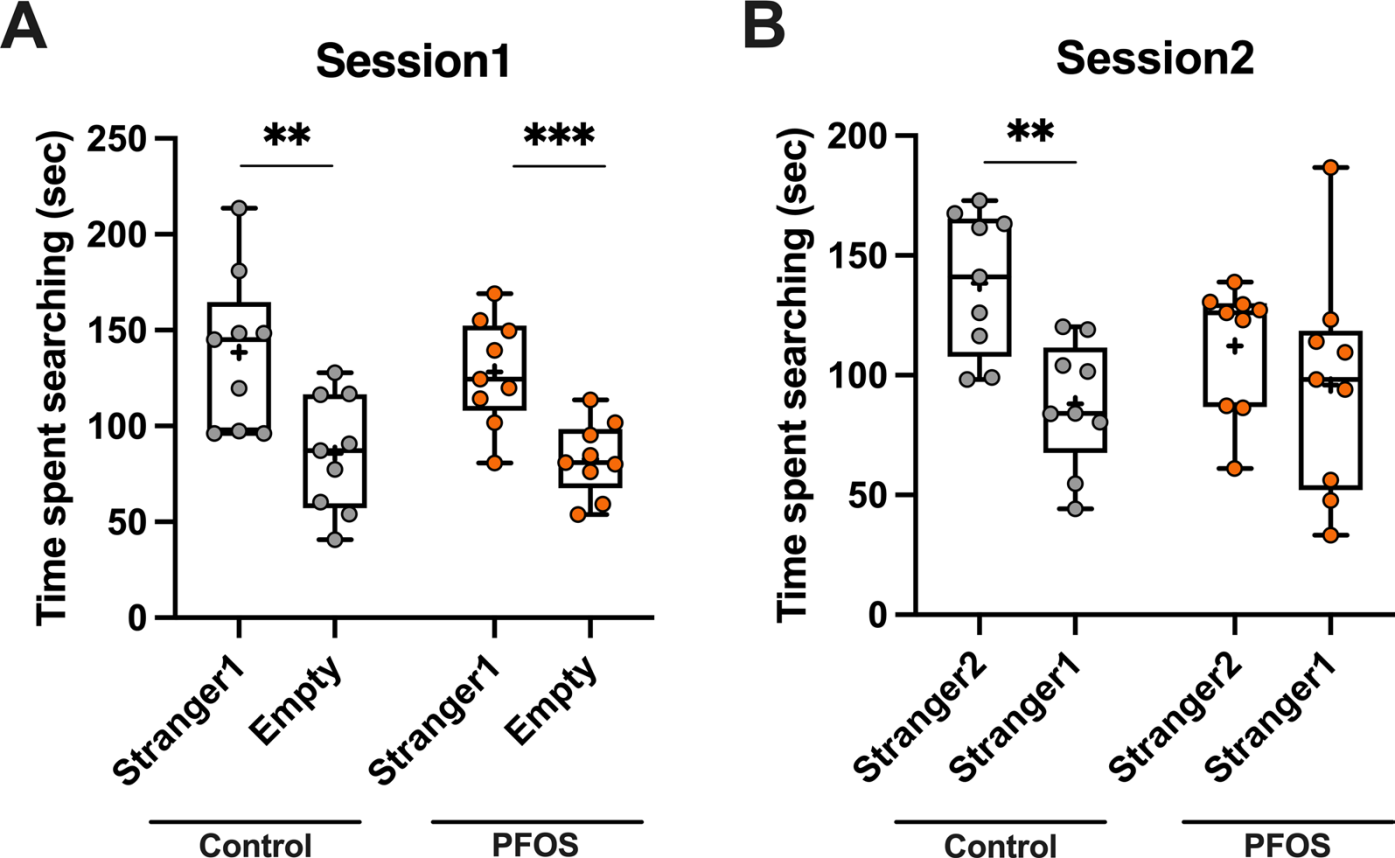
The effects of lactational PFOS exposure on social recognition in middle-aged period. In session 1 of the three-chamber social interaction test, both groups spent longer time on interacting with a novel mouse (stranger 1) than an empty box (**A**). In session 2, control mice spent longer time on interacting with another novel mouse (stranger 2) than the familiar mouse (stranger 1). However, there was no difference in interaction time between stranger 1 and 2 in PFOS group (**B**). ** and *** indicate *p* < 0.01 and 0.001, respectively, by unpaired *t*-test. Data are expressed as the mean $\pm$ SEM. *n* = 9 mice in each group

Three-chamber social interaction test was conducted to investigate the lactational PFOS effects on social behavior. In the session 1, both groups spent longer time on approaching to stranger 1 than to an empty box (control: [stranger 1] 138.4 $\pm$ 13.6 s, [empty] 85.7 $\pm$ 10.16 s, *t* = 3.106, *p* = 0.007, PFOS: [stranger 1] 128.2 $\pm$ 9.284 s, [empty] 82.84 $\pm$ 6.367 s, *t* = 4.032, *p* = 0.001, Fig. 5A). In the session 2, the control mice spent longer time on interacting with stranger 2 than stranger 1 ([stranger 2] 138.5 $\pm$ 9.86 s, [stranger 1] 88.02 $\pm$ 8.79 s, *t* = 3.82, *p* = 0.0015, Fig. 5B). However, there was no difference in the interaction time between stranger 1 and 2 in the PFOS mice ([stranger 2] 112.2 $\pm$ 8.98 s, [stranger 1] 95.91 $\pm$ 15.51 s, *t* = 0.91, *p* = 0.3763, Fig. 5B), suggesting that the lactational PFOS exposure may have a long-lasting effect on social recognition in middle-aged period.

### Expression of Alzheimer’s disease-related proteins in the hippocampus

**Fig. 6:**
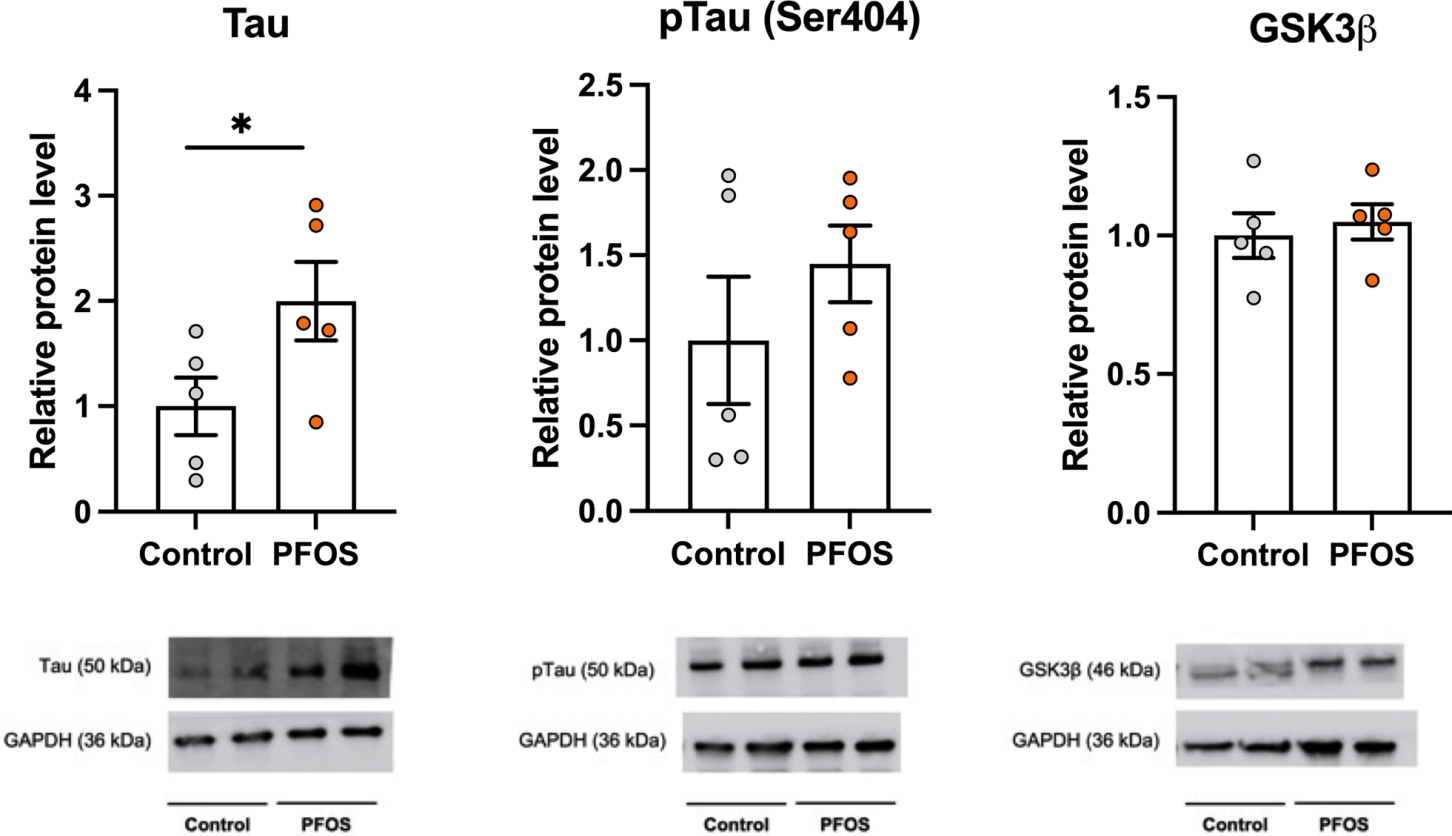
Expression level of proteins related to Alzheimer’s disease. The expression of tau protein in the hippocampus was increased in PFOS mice compared to control mice. There was no change in the expression of phosphorylated tau (Ser404) and GSK3 $\beta$ in PFOS group. * indicates *p* < 0.05 by Mann–Whitney test. Data are expressed as the mean $\pm$ SEM. *n* = 5 mice in each group

Western blot analysis was carried out to examine the expression level of proteins related to Alzheimer’s disease pathogenesis. The result revealed the significant increase in tau protein in the hippocampus of aged PFOS mice compared to the control mice ([control] 1.00 $\pm$ 0.27, [PFOS] 1.998 $\pm$ 0.37, Mann–Whitney *U* = 3, *p* = 0.04, Fig. 6). There was no significant difference in the expressions of glycogen synthase kinase-3 beta (GSK3 $\beta$) protein and phosphorylated tau (Ser404) protein in the PFOS mice (GSK3 $\beta$: [control] 1.00 $\pm$ 0.08, [PFOS] 1.05 $\pm$ 0.06, Mann–Whitney *U* = 9, *p* = 0.5476, Ser404: [control] 1.00 $\pm$ 0.37, [PFOS] 1.449 $\pm$ 0.23, Mann–Whitney *U* = 9, *p* = 0.5476, Fig. 6).

## Discussion

The present study examined the long-lasting effects of early-life exposure to PFOS on behavioral alteration of middle-aged mice. We found the mild effects on social recognition and the increased expression of tau protein in the hippocampus of middle-aged mice who were exposed to PFOS though the breast milk during the lactation.

As learning and memory weigh heavily in cognition, their degradation is getting prominent as aging [19]. Nevertheless, we found no change in learning ability in the middle-aged PFOS group in the visual discrimination test (Fig. 3A). We previously examined the effects of lactational PFOS exposure on learning of young adult mice using the same protocol and found that PFOS mice show the significant impairment in learning for the test duration of nine days [7]. Their percentage of correct answer started from 50% on Day 1 and ended up with 70% on Day 9, which is almost the same as what we observed in the present study for both groups (Fig. 3). Remarkably, the learning efficiency of control mice has drastically attenuated in middle-aged period compared to young adulthood [7]. In the previous study, they already reached 80–90% of correct answer rate on Day 6 and kept it for the last three days of the experiment (showing a “plateau” effect). On the other hand, they did not show a plateau in the learning curve and the improvement in their performance was enormously slow (Fig. 3). If we consider this change as an aging effect, it comes hard to interpret the result of PFOS mice showing no change in learning efficiency over time. This may indicate that the PFOS effects on learning still persist in middle-aged period. However, the present result cannot tell without any significant differences from the control group whose performance may be masked by aging effect. In the meantime, PFOS mice displayed the impaired spatial memory in object location test, which is consistent with the young-adult mice [7]. However, the result of object recognition memory conflicts with what we observed in the young-adult mice which displayed the disruption in discriminating the replaced novel object [7].

Not only learning and memory, but aging also affects social and affective processing [20]. In terms of affective domains, the developing PFOS exposure had no effects on an anxiety-like behavior in middle age (Fig. 4), which is consistent with our previous observation in young adults [10]. On one hand, the results from three-chamber social interaction test tells a different PFOS effect between younger age and older age (Fig. 5). In the session 1, both control and PFOS mice preferred interacting with animate object (stranger 1) to exploring an empty box (Fig. 5A). This indicates that the curiosity for the stranger is preserved in aged PFOS mice. However, in the session 2, whereas the control mice showed a significant increase in interaction with another stranger who was introduced to the chamber environment (stranger 2) compared to the familiar one (stranger 1), PFOS mice spent almost the same time on interacting with stranger 2 as stranger 1 (Fig. 5B). These results may suggest that there is a difficulty in middle-aged PFOS mice in distinguishing individuals. Given that this effect was not previously observed in young adults [10], this could be related with age-related mental disabilities such as dementia, in which patients are unable to memorize and recognize individuals following the cognitive dysfunction [21]. The past studies have presented a correlation between PFOS exposure and Alzheimer’s disease, which accounts for approximately 60–80% of dementia [11–14]. In a prodromal stage of Alzheimer’s disease, the atrophy of the anterior hippocampus, equivalent to the ventral hippocampus in mice, is primarily observed [22]. Social discriminatory behavior is maintained by the activation of ventral hippocampal neurons projecting to the nucleus accumbens, followed by calcium-induced firing of ventral hippocampal neurons [23]. The effects of perinatal PFOS exposure on calcium-dependent signaling in rat’s hippocampus is shown by inhibition of LTP and the change in gene expression such as Ca^2+^/calmodulin-dependent kinase II alpha and calmodulin after development [9, 24]. These electrophysiological and molecular changes could happen in younger age, however, we observed no change in social recognition in young adulthood [10]. In older age, the accumulation of such PFOS neurotoxicity from early life may eventually make the ventral hippocampal neurons vulnerable to neurodegeneration.

Hyperphosphorylation of tau, an intracellular microtubule-binding protein, is considered as one of the major hallmarks of Alzheimer’s disease [25]. In such pathological conditions, the binding affinity of tau to microtubules is weakened, leading to the disruption of axonal transport ([25]). GSK3 $\beta$ plays an important role in phosphorylation of tau protein at specific sites such as Ser404, Ser202, Thr181, Ser199 [26]. There were no changes in GSK3 $\beta$ and phosphorylated tau at Ser404 (Fig. 6), indicating phosphorylation pathway of tau protein may not be affected by the PFOS exposure. For the limitation of the present study, phosphorylation of GSK3 $\beta$ could have been examined as GSK3 $\beta$ activation ([27]). There is a possibility that PFOS may inhibit phosphorylation of GSK3 $\beta$, leading to no change in GSK3 $\beta$ protein levels. The other phosphorylation sites of tau protein should be investigated in the further studies as well. In the present study, we found a significant increase in tau protein in the hippocampus of middle-aged PFOS mice (Fig. 6), which is consistent with the previous study using both pre- and postnatal PFOS exposure rat model [13]. The increase in total tau causes neuronal damage, whereas hyperphosphorylation of tau causes neurofibrillary tangles [28, 29]. Accordingly, it is likely that the early-life PFOS exposure in the present study induces neuronal damage in the hippocampus later in life. Alternatively, no change in tau phosphorylation pathway may account for the mild impairments in cognitive behavior observed in the present study. The other study using the neonatal PFOS exposure model reported that an elevation of tau expression in the brain is associated with the altered response of the cholinergic system in adulthood [30]. The subsequent effects on synaptic plasticity and cognitive function in aged PFOS models should be warranted by the further investigations.

Although we used older model compared to the previous studies [13, 14], one year old is still considered as “middle age”, equivalent to early-middle 40 s in human age [31]. As Alzheimer’s disease most commonly affects older adults aged > 65 years [32], the current model is not completely appropriate to assess age-related disease. Yet, it can also happen in younger people in their 30 s or 40 s, known as early-onset Alzheimer’s disease. The symptoms and progresses of early-onset Alzheimer’s disease is the same as late-onset Alzheimer’s disease in older people [33]. The pathogenesis is also common in early- and late-onset Alzheimer’s disease, accumulation of amyloid-$\beta$ plaques and tau tangles [33]. Therefore, the present finding could account for the PFOS effects on early-onset Alzheimer’s disease at least. Further studies are needed with more aged models at the age of 18–24 months to monitor the change in cognition and biomarkers for the disease.

## Conclusions

The present study revealed that an early-life exposure to PFOS remains a mild cognitive impairment, in particular social recognition, in middle-aged period. Even with a mild impairment, taken together with previous reports, the current findings call attention to the certain risks for neurodegenerative diseases in aging population with an early history of PFOS exposure.

## Data Availability

The datasets used and/or analyzed during the current study are available from the corresponding author on reasonable request.

## Abbreviations

PFOS: Perfluorooctane sulfonate
PFAS: Per- and polyfluoroalkyl substances
PD: Postnatal day
SEM: Standard error of the mean
GSK3$\beta$: Glycogen synthase kinase-3 beta
